# Efficiency and Compatibility of Selected Alkoxysilanes on Porous Carbonate and Silicate Stones

**DOI:** 10.3390/ma12010156

**Published:** 2019-01-06

**Authors:** Matea Ban, Elisabeth Mascha, Johannes Weber, Andreas Rohatsch, José Delgado Rodrigues

**Affiliations:** 1 Institute of Geotechnics, Research Centre of Engineering Geology, Vienna University of Technology, 1040 Vienna, Austria; andreas.rohatsch@tuwien.ac.at; 2 Institute of Art and Technology, Conservation Science, University of Applied Arts Vienna, 1010 Vienna, Austria; elisabeth.mascha@uni-ak.ac.at (E.M.); johannes.weber@uni-ak.ac.at (J.W.); 3 National Laboratory for Civil Engineering Lisbon, 1700-066 Lisbon, Portugal; delgado@lnec.pt

**Keywords:** stone consolidants, tetraethyl-orthosilicate, ethyl silicate, TEOS, alkyl-trialkoxysilane, efficiency, compatibility, natural stone, mechanical testing, scanning electron microscopy

## Abstract

This study compares the consolidation efficiency and compatibility of three selected alkoxysilanes on two porous carbonate and silicate substrates. Emphasis was given to artificially induced microstructural defects and subsequent restoration of mechanical and physical properties. Two newly engineered formulations (1) a TiO_2_ modified tetraethyl-orthosilicate in isopropanol with 70% active content and (2) a TiO_2_ modified alkyl-trialkoxysilane in isopropanol with 75% active content were compared to a commercial product (3), a solvent free tetraethyl-orthosilicate with 99% active content. Treatments were evaluated by scanning electron microscopy, mercury intrusion porosimetry, colour impact and their effect on dynamic modulus of elasticity, splitting tensile- and flexural strengths, capillary water absorption and water vapour permeability. A key outcome was that mechanical strength gain induced by treatments is primarily governed by a stone’s texture and microstructure, and secondarily by the gel deposition rate of consolidants. Likewise, the kinetics of the gel-forming reaction during curing is dependent not only on the product but also on the substrate. Therefore, the moisture related properties and the visual impact develop during time. There is no general trend on how it evolves in time, which can lead to incorrect interpretations of treatment compatibility. The results prove that wide-ranging treatment performance is obtained when applying the same products on different substrates.

## 1. Introduction

A wide variety of natural stones are used as building materials; they exhibit a correspondingly wide range of chemical, physical and mechanical properties. When exposed to outdoor environments, they undergo changes that range from surface decay and erosion to deep structural disintegration [1]. This decay features often display complex morphological patterns and may necessitate the stone’s consolidation to reinstate the lost cohesion. While the assessment of the success of laboratory consolidation treatments of selected test specimens is not simple and depends on a variety of factors, the assessment of on-site treatments of naturally weathered stones under real conditions is even harder and sometimes unpredictable. Every characteristic of the stone as well as the treatment procedures may have a major impact on the efficiency and compatibility of the treatment [2], and by varying one of them, the results might change drastically.

When testing a newly engineered consolidant, the basic conditions of the product application need to be kept simple and reproducible to test the properties of the consolidant in a systematic manner, following a tailored specific protocol. Three main alternatives may be followed in the testing process: (i) to use homogeneous, fresh stone specimens; (ii) to test on naturally aged and (iii) on artificially aged specimens. The first option reduces the number of variables in the process and makes results easier to compare, while the second one tends to increase heterogeneity and dispersion of results. The second alternative is usually supported with the argument that it is more representative of natural conditions. Using samples taken from naturally aged stone would be a good option, but rarely are enough of such samples available for extensive laboratory testing and, therefore, artificially aged samples were used along this study. Most test methods are appropriate for homogeneous specimens of reasonable dimensions; therefore, artificially aged specimens rarely entirely fulfil such requirements and the interpretation of results becomes more difficult. To overcome this important limitation, some authors prefer to use porous stone varieties of similar mineralogical composition in their sound state [3,4].

Artificial aging induces microstructural defects intended to mimic a certain type of natural decay process, thus, it allows the study of the solely properties of a consolidant, and its ability to improve the mechanical and physical properties of a stone. To prepare artificially decayed samples, e.g., thermal treatment of stone specimens [5], and freeze-thaw cycles [6] have been used. Thermal treatment induces micro cracks that correspond to a frequent type of de-cohesion formed by weathering on-site; they reduce the stone soundness by decreasing its mechanical strength and increasing its porosity and rate of capillary absorption [7]. As for freeze-thaw ageing, some stone varieties require many cycles before a microstructural defect is induced and others break prematurely due to the formation of larger cracks, which makes this method not adequate for large-scale testing purposes, especially mechanical testing [8]. Other laboratory ageing methods e.g., with salt crystallization or acid attack produce a pronounced gradient of degradation, which may lead to results difficult to interpret, even when the obtained decay pattern appears to be close to reality. Thermal cycles on the other hand are likely to produce a homogeneous decay pattern through the full diameter of the stone, a prerequisite for testing the macro properties according to most standard natural stone test methods [9].

Whatever aging method is used, the aged samples will never fully mimic reality. Consequently, the option for one or another strategy mostly depends on the available methods and means, and a best fit scenario to test the given hypotheses.

A closer look on the meaning of the terms “efficiency” and “compatibility” of stone consolidants is useful to understand how to assess them. The primary function of a stone consolidant is to re-establish the cohesion between grains thus restoring the mechanical properties of the weathered stone material [10]. Only in this way, the product can be assumed to fulfil its primary function of enhancing a stone’s strength. Therefore, in order to assess its efficiency, the mechanical properties of the specimen before and after the treatment need to be evaluated. The first issue that arises hereby is the definition of the degree to which the mechanical strength needs to be increased in respect to a chosen starting condition, i.e., sound or aged. There are no general rules and only the notion except the general consensus that the treated stone should not be excessively stiffer than the substrate [11,12]. The reasons for the variation in the mechanical strength need to be studied, but insofar as our knowledge of the mechanisms involved in the strength increase due to the treatment is incomplete and vague, validation of test results remains empirical. As an example, it is accepted that increasing the mechanical strength by only a few percent—and this effect was observed in many studies—may be enough when certain decay patterns are in question [13,14].

In the laboratory testing routine, a few techniques including drilling resistance, tensile strength testing, and measurement of ultrasound pulse velocity are employed to evaluate the efficiency of consolidants [15,16,17,18,19,20]. In addition, various types of tensile test, which include the pull-off adhesive, splitting tensile- or flexural strength tests, are indicators of microstructural defects and thus are apt to describe in the most effective way the action the consolidant exerts on a stone fabric [21]. Even though these methods provide better insights into the effect of a treatment, it must be kept in mind that the applied stresses do not correspond to the micro- and macro mechanical stresses that a material has to bear on-site where e.g., shear stresses parallel to the surface of exposure may play a major role.

A further technique used to assess the efficiency of a consolidation treatment is the determination of the dynamic modulus of elasticity as described by the fundamental resonance frequency and ultrasound pulse velocity. This is an accurate method capable of describing indirectly the closure of pores and cracks. It can give a bulk indication of the amount of consolidant deposited in the stone. Nevertheless, when a stone accommodates a large amount of a weakly strengthening consolidant, the efficiency may still be poor, which is why a combination of different tests is recommended to assess the treatment’s efficiency. Despite the variety of test methods currently available, a systematic protocol to evaluate the efficiency of consolidants—with a multiscale approach—has yet to be defined in the field of architectural conservation science.

The secondary role of a consolidant is its ability to restore the physical properties of the weathered fabric without potential side effects [22]. This is particularly important for the compatibility with the substrate, a broad term comprising issues such as the chemical affinity and the impact on physical parameters relevant to identify and quantify the potential damages caused by the treatment [23,24]. The physical properties frequently used are porosity and moisture related properties, such as water vapour permeability, pore size distribution, and the drying rate of treated vs. untreated stone.

Further means to study compatibility and predict harmfulness may comprise scanning electron microscopy (SEM)-techniques on polished sections of treated specimens. This approach yields information useful to understand both efficiency and compatibility criteria. It can give qualitative insights into the treatment by showing the adhesion to the substrate, bridging capacities, consolidant shrinkage, and the in-depth distribution of the solid consolidant residue in the substrate pore system. Quantitative image analysis may show the depth and degree of pore filling [25,26,27]. It is of utmost importance to correlate the macro properties of treated specimens with the SEM observations related to the penetration depth and spatial distribution of the consolidant. SEM, for example, is capable of detecting over-consolidation of surface areas, which could explain the results of a drilling resistance measurement.

Compatibility also comprises the visual aspects of a consolidated stone. Well established techniques are used to quantify colour changes and gloss induced by a treatment. It should be kept in mind, however, that these types of changes may regress in time, thus reducing or eliminating their negative impact [28]. Far too seldom have the various techniques employed to assess compatibility been correlated with each other, and still much rarer are the cases where compatibility is comprehensively assessed in a multiparametric approach.

A group of consolidants that has been in use for decades belongs to the family of tetraethyl-orthosilicates (TEOS). Based on the general formula of Si(OR)_4_ the products have been modified for different purposes. They were engineered to be water repellent [29,30], have photocatalytic- [31,32] or elastified properties [33] or to have a coupling agent to better adhere e.g., to a carbonate substrate [34]. The gel deposition rate, or the solid content after curing, plays a crucial role in inducing mechanical improvement, and several products are on the market with different gel deposition rates [35]. These modifications are supposed to have impact on the efficiency and compatibility of the consolidation treatment.

This study aims at assessing the effectiveness and compatibility of three different alkoxysilanes applied on one carbonate and one silicate substrates. Strength increase and physical changes induced by the treatments are compared. Two consolidants were newly engineered in the framework of the European project Nano-Cathedral—short for—“Nanomaterials for conservation of European architectural heritage developed by research on characteristic lithotypes” (Grant Agreement No. 646178, Call NMP-21-2014: Materials-based solutions for protection or preservation of European cultural heritage). The products were developed for a large-scale technological applicability by two SME partners. The details of the synthesis route are protected by a non-disclosure agreement. The two novel products are based on nano-TiO_2_ particles linked to ethylic esters of silicic acid in isopropanol. The first product NC-25C is a tetraethyl-orthosilicate in isopropanol, doped with nano-TiO_2_ to have photocatalytic activity. It has 70% active content and contains 10^−2^ ppm TiO_2_ with less than 100 nm in size (generally between 5 and 40 nm, with the highest amount average in size between 10 and 15 nm). The second product NC-27CP is an alkyl-trialkoxysilane in isopropanol with 75% active content and it is containing 10^−2^ ppm TiO_2_ particles with the same dimensions as described above. The products typical Si–O–C chain was modified with organic groups to obtain higher elasticity by forming Si–O–(CH_2_)_n_–O–Si. Nano TiO_2_ is added to reduce the shrinkage of the consolidating gel and to confer photocatalytic activity to the stone surface. The spherical shaped TiO_2_ nano particles occur in two crystal phases, namely anatase (78.8%) and rutile (21.2%). Although the modification of consolidants with nano-TiO_2_ has been the subject of several laboratory studies [36,37], such products are not yet available on the market in large quantities. The potential self-cleaning ability conferred by the nano-TiO_2_ particles is an added functionality to the consolidants, but it was not integrated in the research work reported here. Both TiO_2_ containing products were developed by the Italian company Chem Spec Srl in the framework of the EU-funded *Nano-Cathedral* project. The supplier of the nanoparticle is the Spanish-based industry Tecnan (Technologia Navarra de Nanoproductos, S.L.). These products were selected due to their higher silica gel content when compared to the common commercial products. In principle, higher silica gel contents should result in advanced mechanical consolidation properties. The quantity of the silica gel deposited in the stone fabric after curing can be varied by the mixing ratio of monomer and oligomer molecules in the product, that is to say, the smaller the molecules, the lower the gel deposition rate. Further possible modifications include the addition of catalysts that influence the reactivity of the product, and the addition of solvents. In this way the products can be customized for varying substrates and decay phenomena. The product used as a reference in the present study, traded under the name KSE 300, is produced by Remmers (Löningen, Germany); it is the most widely used stone consolidant in Central Europe. This is a solvent-free tetraethyl-orthosilicate with a gel deposition rate of approximately 30%, which corresponds to 300 g of solid silica content per litre of consolidants.

The results of this study show that treatments’ effectiveness is primarily governed by the texture and microstructure of the natural stone and secondarily by the solid content after curing. While in general all studied alkoxysilanes showed to have a certain consolidating effect, the same product gives different results in terms of strength and physical properties when applied on different substrates. Moreover, reaction kinetics is also stone dependent and the time for curing through hydrolysis and condensation differs between the substrates. Because of the curing time, all moisture related properties and colour changes are time-dependent values. In respect to the substrate, the evolution of those values in time follows no general trend and they even have opposing directions varying in absolute and relative magnitude. These results lead to the conclusion that the interpretation of treatments compatibility has to be done with care; especially as the speed of the gel deposit reaction is not only temperature and humidity but also substrate dependent.

## 2. Materials and Methods

### 2.1. Petrographic Characterisation

**Figure 1:**
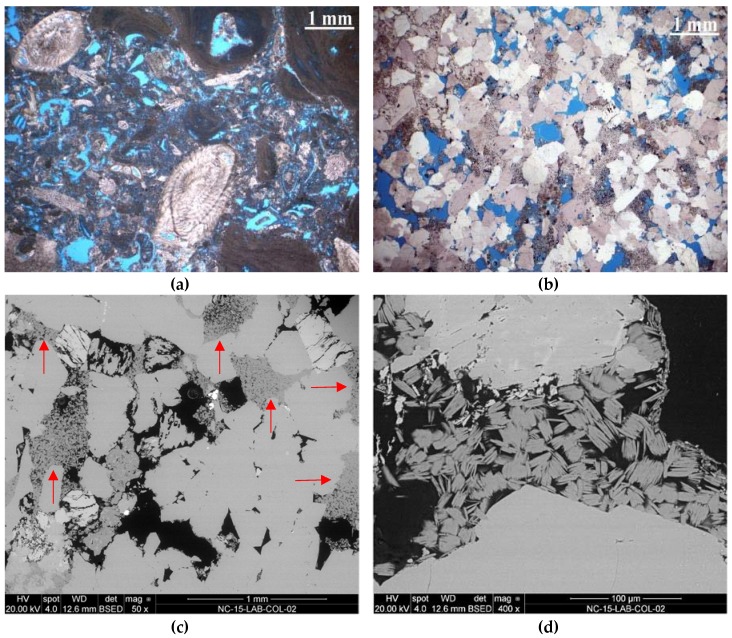
Microphotos of thin sections recorded with a polarised-light microscope (PLM): (**a**) *St. Margarethen Limestone* with different microfossils such as foraminifers, coralline red algae and debris of echinites; (**b**) *Schlaitdorf Sandstone*: a coarse-grained quartz arenite with kaolinite and sparitic dolomite; Microphotos of cross sections recorded with a scanning electron microscope (SEM): (**c**) occurrence of kaolinite (red arrows) up to 15% in *Schlaitdorf Sandstone* indicating its even distribution throughout the fabric; (**d**) detail of kaolinite in the intergranular pore space between quartz and feldspar.

Two porous lithotypes of historic monumental importance, one silicate and one carbonate, were selected as testing substrates:

*Schlaitdorf Sandstone*, historically quarried in Baden-Württemberg, Germany, is a coarse-grained siliciclastic arenite, classified after R.L. Folk [38,39] as subarkose from the Upper Triassic, with a light to reddish colour and an average grain size of about 0.5 mm (Figure 1b). Quartz, feldspar and rock fragments are roughly equigranular, vary from subangular to rounded and are tightly intergrown. Its texture is mostly homogeneous without any marked layering. The authigenic minerals sum up to 15% and consist of sparitic dolomite, microcrystalline quartz, kaolinite and traces of illite, partly filling the intergranular pores [40] (p. 56). The typical pore size ranges from 0.2 to 1 mm and the effective open porosity measured by Hg-porosimetry is about 15.4%. *Schlaitdorf Sandstone* has been used as construction material for prominent buildings, such as the cathedrals of Cologne and Münster [41]. The main deterioration phenomena affecting this lithotype are sanding, flaking and scaling [42].

*St. Margarethen Limestone* is a porous calcareous arenite, quarried in the Rust Hills of Burgenland (Eastern Austria). It is a biogenic detritic sedimentary rock from the Miocene (Langhian) age [43]. It can be classified as a biosparite and grainstone [44,45]. Its colour ranges from yellow-brownish to light grey. This lithotype mainly consists of small fragments of coralline red algae, foraminifers, fragments of bryozoans and echinoids (Figure 1a). Calcite is the dominating mineral, with traces of siliciclastic detritus, mainly quartz and muscovite. The components are cemented with a fine-grained, early diagenetic dogtooth-calcite. Pores and voids range from a few micrometres to millimetre range, and the total open porosity measured by Hg-porosimetry is about 20.7%. *St. Margarethen Limestone* has been used as a construction and sculptural stone from Roman times until today [46]. The main deterioration phenomena affecting this lithotype are granular disintegration, formation of black crusts, scaling, sanding and bursting [47]. This lithotype was used for the restoration of the St. Stephens Cathedral in Vienna since 1845 and in countless monuments in Vienna (e.g., Vienna Town Hall, Palace and Gardens of Schönbrunn, historic St. Marx Cemetery, etc.) and elsewhere in Eastern Austria [48].

### 2.2. Artificial Ageing by Thermal Treatment

Stone specimens of defined geometry and dimensions as required for standardised stone test methods were exposed to a cyclic heat treatment in order to create relevant microtextural defects. The heating gradient was set to a rate of 40 °C/min with a peak temperature of 580 °C for one hour. The cooling phase to room temperature took 24 h before the same procedure was applied. In total three such cycles of heating and cooling were applied. These parameters had been established by a systematic experimental study [9] to ensure the formation of micro fissures throughout the complete sample thickness, which were expected to significantly alter the modulus of elasticity and the water absorption coefficient. In principle, the optimum residence times and peak temperature for each lithotype depend on the dimensions and shape of the specimen and had to be adapted accordingly. To remove all loose dust from the specimens, after the artificial ageing the specimens were washed and dried to constant weight.

### 2.3. Consolidation Treatment

As pre-conditioning before the application of consolidants, specimens were stored in a container at 70% RH for one week. Then, they were placed on a metal grid with their bottom in contact with the consolidant for one hour, to enable absorption by capillary suction. Within that time, full saturation was achieved in all cases. After the treatment with the consolidants, the specimens were weighed and placed in an airtight container. After 24 h the same treatment procedure was repeated. For the following three days, the consolidated specimens were placed in a sealed container next to cups filled with the consolidation products in order to saturate the atmosphere with the corresponding solvents. To control the backwards migration of the consolidant in the stone specimens by preventing overly rapid evaporation, the box was opened gradually for a period of one week, during which the stone was additionally covered with a plastic foil. In the container the specimens were placed on a grid to assure drying from all surfaces. To observe the reaction and evaporation process, the weight of each consolidated specimen was recorded until constant mass. However, it must be emphasised that mass stability might not necessarily indicate complete reaction of the system, since several products proved still water-repellent beyond the attainment of gravimetric stability, a condition that is typically absent form fully reacted consolidant. Most tested properties of the treated stones are dependent on the consolidant curing state, namely the products’ hydrolysis and condensation degree, both implying mass change. The elapsed curing time before testing is indicated in the corresponding tables and diagrams.

### 2.4. Test Methods for Determining the Efficiency and Compatibility of Consolidants

The artificially aged and subsequently treated specimens were compared to untreated artificially aged and untreated sound specimens. The following test protocol was applied to evaluate those conditions:

1. For the preparation of polished cross sections, laboratory treated specimens with dimensions 50 mm × 50 mm × 50 mm were vacuum impregnated with blue stained epoxy resin and cut perpendicular to the consolidated surface. The section size ensured that treatments which had reached the centre of the treated body could be traced by the analysis. The slices were then polished and examined by SEM. The instrument used was a field-emission scanning electron microscope of the type *FEI Quanta 250 FEG*. The working mode was low vacuum at 20 kV acceleration voltage. Contrast and brightness of the backscattered electron (BSE) images were adjusted to visualize the consolidant in the pores by distinct grey values. For visualisation purpose and image analysis, post processing of the micrographs was done with *Adobe Photoshop* by false-colour-mapping the silica gel inside the stone fabric. Furthermore, when calculations of e.g., the penetration depth to filling degree was to be obtained, the software *ImageJ* proved to be a valuable tool for such purposes.
2. Mercury intrusion porosimetry (MIP) was performed with a Porosimeter *Porotec Pascal 140/440*. Changes in the pore radii distribution and open porosity determined by Hg-intrusion for the different conditions (sound, artificially aged and consolidated) were studied.
3. The dynamic modulus of elasticity was determined with the longitudinal resonance frequency of an ultrasound signal in transmission, according to EN 14146 [49]. Prismatic specimens with dimensions 10 mm × 10 mm × 40 mm were used and mean values of three specimens were computed. The small specimen size was selected to ensure the consolidation of the entire body. The test was performed by an ultrasound pulse generator (*CONOSONIC C2-GS*), a pair of transducers (*UP-DW*), a clamping and pressure device for specimen’s assembly as well as and a notebook preinstalled with the *Light House DW* software, developed by *Geotron-Elektronik*, Germany. The UP-DW piezoelectric transducers, operating at a frequency band from 1 to 100 kHz, are specifically manufactured to determine the materials elastic parameter (DW stands for “dehnwelle” and translated from German it means “extensional wave”). This device is equipped with a built-in algorithm that calculates the longitudinal-, transverse-, surface- and extensional waves as well as E- and G Modulus and the Poisson’s ratio. However, the principle on how to obtain the dynamic modulus of elasticity (Ed_L_) determined through the longitudinal fundamental resonance frequency (F_L_) is given by Equation (1):
  *Ed_L_* = 4 × 10^−6^*·l*^2^*·*F_L_^2^*·ρ·T* (1)
  whereby (*l*) represents the specimens length and (*ρ*) the stones apparent density. As in our case the width of the specimen is four times its length, the correction factor (*T*) can be assumed to be 1 in which case the equation is simplified to Equation (2):
  *Ed_L_* = 4 × 10^−6^*·l*^2^*·*F_L_^2^*·ρ* (2)
  (F_L_) was recorded when the deviation of the measured fundamental resonance frequency stayed in a range of ±60 Hz, three times in a row. The dynamic modulus of elasticity is reported in GPa or kN/mm^2^. As for all non-destructive tests, measurements could be performed on the same specimen before and after treatment.
4. Splitting tensile strength was determined following the recommendations of ASTM D 3967-08 [50]. The electro-mechanical tension and compression-testing machine was a 150 kN *Instron Model 4206*, developed by *Instron GmbH*, in Germany. The apparatus consisted of a flat bearing block at the bottom and, to reduce the contact stresses, a curved bearing block on the top. Bearing strips with 0.6 mm thickness were used to reduce high stress concentrations. The loading rate was 100 N/s. 16 specimens per lithotype and condition (sound, aged and consolidated) were tested, each 60 mm in diameter and 30 mm in thickness. For the aged stone specimens and the reference product KSE 300, 10 out of the 16 specimens were tested in the frame of two master theses [51,52]. The test was executed in the direction perpendicular to the bedding plane, which was assessed through ultrasound pulse velocity. For the latter purposes, the frequency for both lithotypes was set to 80 kHz and the amplitude was adjusted according to the samples damping. Specimens were measured without a coupling medium. The splitting tensile strength was calculated with Equation (3) and is here reported in N/mm^2^.
  *σ_t_* = 2 × π^−1^·*P*·*L*^−1^·*D*^−1^ (3)
  (*P*) is the maximum applied load indicated by the testing machine in newton [N], (*L*) the thickness, and (*D*) the diameter of the specimen, in mm.
5. The three-point flexural strength was determined according to EN 12372 [53] with the load increased uniformly at a rate of 0.25 ± 0.05 MPa/s (or 41.67 N/s recalculated for the given dimensions) until the specimen broke. 10 specimens with 25 mm × 50 mm × 150 mm were tested, whereby the distance between the supporting rollers was 125 mm. The tests were performed with an electronic spindle-drive testing machine of the type *Testomeric Quicktester 100 kN* and evaluated by the *Test & Motion* software developed by *DOLI Elektronik GmbH*, Germany. The test was carried out in the direction perpendicular to the bedding plane, which was assessed through ultrasound pulse velocity. The flexural strength was calculated according to the following Equation (4):
  *R_tf_* = 1.5·*F*·*l*·*b*^−1^·*h*^−2^ (4)
  where (*F*) is the breaking load in newton, (*l*) the distance between the supporting rollers, (*b*) the width- and (*h*) the thickness of specimen adjacent to the plane of fracture, all reported in mm. The results are expressed in MPa or here in N/mm^2^ (1 MPa = 1 N/mm^2^).
6. Water absorption coefficient after one hour was determined according to standard EN 15801 [54] and is reported as kg·m^−2^·h^−0.5^. The test was carried out on three 30 mm × 30 mm × 30 mm specimens per stone and treatment. After a stage of pre-conditioning, samples were placed on water-soaked filter paper (*Ahlstrom-Munktell* laboratory filter paper, wet-strengthen grades) and the absorption of water was monitored gravimetrically. The test was performed on the same specimens before and after treatment.
7. Contact angle of water was determined on the stone surface treated with the water repellent consolidants NC-27CP. Therefore, the *Mobile Surface Analyzer* from *Krüss GmbH*, Germany came to use.
8. Water vapour permeability tests were performed according to EN 15803 [55] using the so-called “wet cup” method with a cup system Type 1 according to the standard. In this case, the cups were filled with water and placed in a climatic chamber at ambient conditions of 23 ± 1 °C and 50 ± 3% RH (*Heraeus Vötsch Klimaprüfschrank VC3, model 4034*). They were weighed every 24 h for one week. The results were plotted as mass change (Δ*m*) against time (*t*) and the slope of the linear section of the curve (*G*, kg·s^−1^) was determined with the software *OriginPro*. (*G*) was further used to determine the water vapour permeance (Equation (5), in kg·m^−2^·s^−1^·Pa^−1^):
  *W_p_* = *G*·*A*^−1^·∆p_v_^−1^ (5)
  where (*A*) represents the specimens surface area in m^2^ and ∆p_v_ the water vapour pressure difference reported as Pa across the test specimen. The water vapour permeability reported in kg·m^−1^·s^−1^·Pa^−1^ was then determined with Equation (6):
  *δ_p_* = *W_p_*·*D* (6)
  where (*D*) represents the average thickness of the test specimens in m. Three specimens per lithotype and treatment with dimensions of 50 mm × 50 mm × 10 mm were tested. The water vapour permeability is reported as the ratio of treated to untreated values.
9. Finally, colour parameters were determined with a *ColorLite sph850* spectrophotometer, according to standard EN 15886 [56]. The output of the measurements is reported as CIE (International Commission on Illumination) L*, a*, b* colour parameters, tested with a D65 illuminant at 10° standard observer with a reflectance spectrum in the range of 400 to 700 nm. ΔE* was reported and describes the metric difference or distance between two colours before and after treatment according to the standards of the International Commission on Illumination. Average (L*), (a*) and (b*) values were used to obtain the total colour difference (ΔE*) between treated (*t*) and untreated (*nt*) measurements with Equation (7).
  (7) $$\Delta E_{t,nt}^{*}={\left[{\left(L_{t}^{*}-L_{nt}^{*}\right)}^{2}+{\left(a_{t}^{*}-a_{nt}^{*}\right)}^{2}+{\left(b_{t}^{*}-b_{nt}^{*}\right)}^{2}\right]}^{0.5}$$
  In the latter equation (ΔL*) corresponds to the lightness difference, (Δa*) to the red/green difference and (Δb*) to the yellow/blue difference of the tested stone specimens. Colour values measured for treated and untreated specimens were performed on sound stones, in order to exclude any possible impact induced by heat treatment. The results were calculated from an average of three measurements obtained at the same spot, with the help of stencils, before and after the treatment.

## 3. Results and Discussion

### 3.1. Spatial Distribution of Consolidants after Curing Assessed by Scanning Electron Microscopy

**Figure 2:**
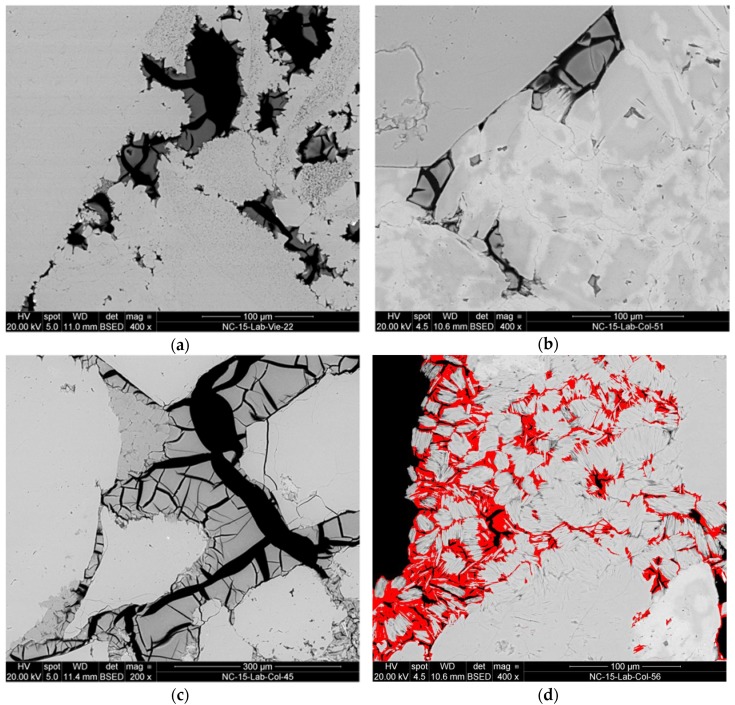
SEM-micrographs displaying some characteristic features of silica gels precipitated from various silicate treatments (gels are clearly visible by their grey value): (**a**) the adhesion of KSE 300 gel to *St. Margarethen Limestone* due to the advantageous topography of the grain boundary, (**b**) worse adhesion of KSE 300 gel to *Schlaitdorf* sandstone, (**c**) the shrinkage of NC-25C gel in intergranular pores of *Schlaitdorf Sandstone,* and (**d**) the interpenetration of the NC-27CP gel (shown in pseudo-colour-imaging) into the clayey matrix of *Schlaitdorf Sandstone.*

The main features studied with SEM were the topographical and morphological appearance of the consolidants and the location of the solid precipitates inside the pore system. This shows the in-depth distribution of the consolidant from the treatment surface inwards and the way it is linked to grains (e.g., coating, or bridging them across pores, the quality of adhesion, and degree of shrinkage). Examples of these features are illustrated in SEM-micrographs shown in Figure 2a–d.

Even if the penetration depth reaches the full depth of the test specimens (50 mm), as observed for all treatments and both stones, differences in the distribution of the solid consolidants are discernible. In the case of *St. Margarethen Limestone* a full and even in-depth-distribution of the solid residues after curing can be observed for treatments NC-25C and NC-27CP, while the solids distributions for KSE 300 can be classified as less homogeneous. In the case of *Schlaitdorf Sandstone* the most homogeneous solids distribution was achieved with NC-27CP while KSE 300 and NC-25C accumulated in the upper parts (approximately 2 cm below the treated surface), as well as on the lateral planes of the samples indicating different rates of back migration upon drying. For both lithotypes, the adhesion of the consolidants to the grains of the substrate is in general not satisfactory, with NC-27CP showing a somewhat better adhesion, followed by KSE 300 and NC-25C treatments. *St. Margarethen Limestone* displays slightly better adhesion because its grain fabric favours the mechanical interlocking (Figure 2a). High shrinkage is visible within all three treatments and for both stones, with KSE 300 and NC-27CP revealing slightly lower shrinkage than NC-25C.

In the case of *St. Margarethen Limestone,* all three products show a high tendency to accumulate in smaller intragranular pores, while only KSE 300 and NC-27CP can be partially observed in the smaller cracks induced by thermal aging. For *Schlaitdorf Sandstone* a slightly better accumulation of all three consolidants within smaller cracks can be determined. A special microtextural feature of *Schlaitdorf Sandstone* was the penetration of the clayey matrix that acts as a filler in the stone while in the consolidated state it might develop properties of a binding medium in the fabric of the sandstone (Figure 2d). Here a better adhesion and a lower shrinkage in the clayey matrix can be observed.

Alkoxysilanes are known to develop cracks inside their gels. Therefore, it is difficult to assess the bridging capacities of these consolidants and report values for their maximal bridging without evaluating this issue in a statistical manner. Due to the drying stresses inside the fabric cracks develop, leaving a plate-like structure of the silica gel as observed in polish cross section. The product manufacturer [57] of classic KSE systems indicates a medium size silica gel plates of approximately 10 µm in size, while Wendler et al. [24] (pp. 47–48, 91) reported bridging capacities to be maximum about 50 µm. In general, the SEM micrographs presented in this study display smaller and larger gel-plates than 50 µm.

It can be concluded that the characteristic properties of the consolidants, namely in-depth distribution, adhesion, shrinkage and bridging capacities, differ for the studied lithotypes. The differences might be explained through the stones texture and topography, while the role played by the stones chemistry cannot be ascertained with these methods and will be the subject of further studies. However, all these properties can be best assessed through SEM of polished cross sections which reveal unambiguous insight into the microstructure, while SEM imaging of fractured surfaces, though more commonly used to visualize consolidants in porous substrates [58,59,60], is more difficult to interpret.

### 3.2. Porometric Characteristics Examined by Mercury Intrusion Porosimetry

**Figure 3:**
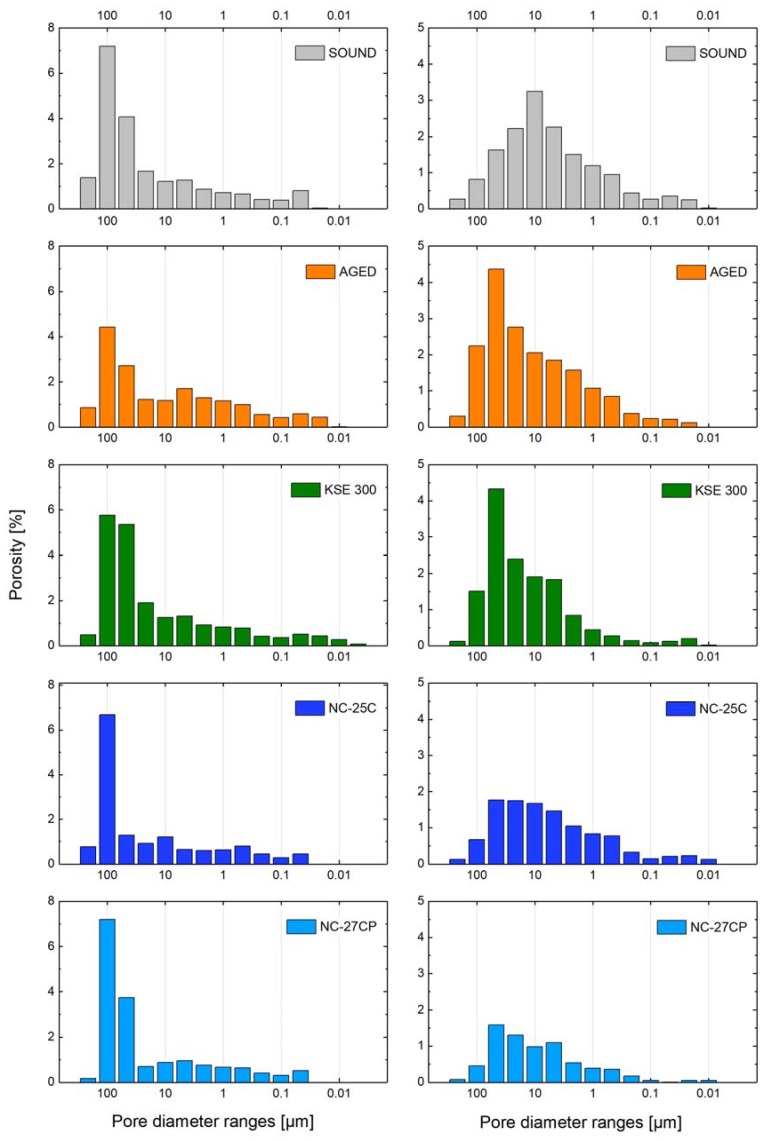
Range of porosimetric parameters (pore radii distribution and open porosity by Hg-intrusion) for the—from top to bottom—sound, aged and consolidated (KSE 300, NC-25C and NC-27CP) conditions determined by mercury intrusion porosimetry; six months after consolidation treatment: values for *St. Margarethen Limestone* (**left side**) and *Schlaitdorf Sandstone* (**right side**).

**Figure 4:**
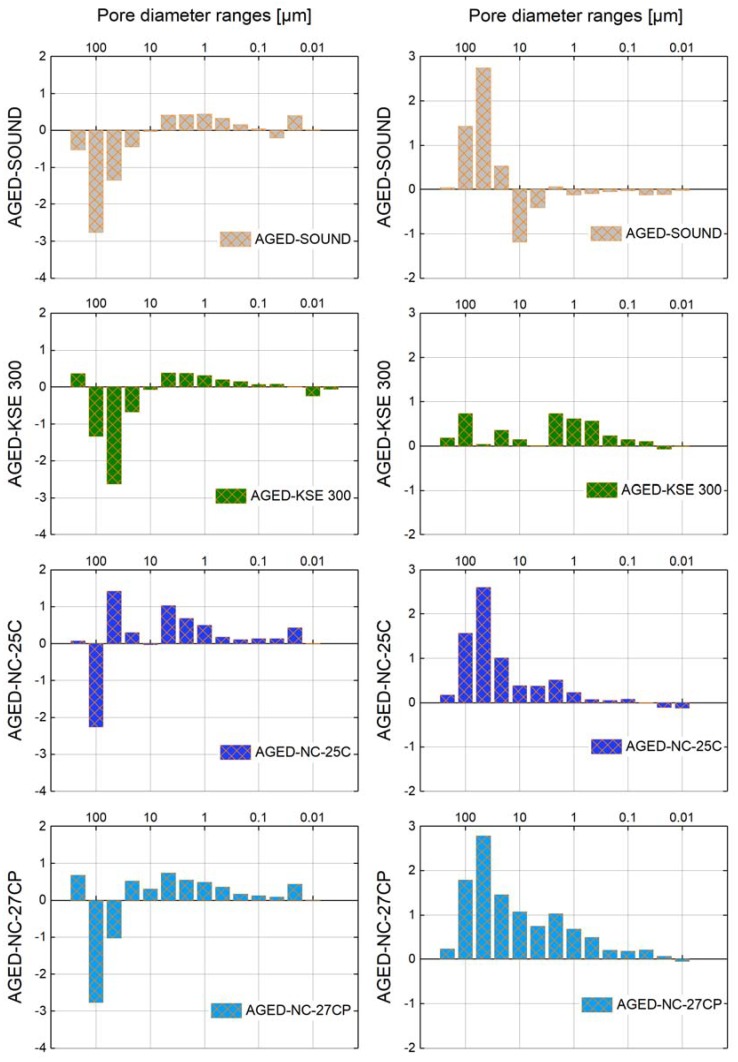
Differences in open porosity for each class of pores determined from the Hg-intrusion porosimetric data. Results for *St. Margarethen Limestone* (**left side**) and *Schlaitdorf Sandstone* (**right side**). To read this comparative graph, from aged to sound conditions, the positive values represent the newly created pores in the respective class. When comparing ages to consolidated conditions, the positive values represent the amount of pores that were filled with the consolidant. Negative values for treated *St. Margarethen Limestone* in the range of pores higher than 10 µm would represent an increase in large pores caused by a treatment, which signify a physical impossibility and are due to the inhomogeneous nature of this lithotype.

Mercury intrusion porosimetry (MIP) was used to study the pore space characteristics in sound, aged and consolidated conditions, thus providing insights into the shift of the pore radii distribution, the typical pores size ranges where consolidants were preferentially deposited, and the possible appearance of secondary porosity created within the consolidating gels (Figure 3 and Figure 4). Furthermore, total pore surface, average pore diameter and total porosity by Hg-intrusion are reported in Table 1. The gel deposition rate or solid content of cured consolidants calculated gravimetrically is reported in Table 2.

Due to its inhomogeneity, the effective total porosity of *St. Margarethen Limestone* shows inconsistent values, which could explain why the specimen in aged condition revealed lower porosity as compared to the sound specimen. This indicates the limitation of the used test method for certain types of stone and the caution that needs to be taken when interpreting these results. *St. Margarethen Limestone* has an inhomogeneous structure and the size of the tested specimen is not fully representative since fossils may reach dimensions up to a few centimetres. Therefore, analysis of both the artificially aged and consolidated conditions, leaves some ambiguity in how to interpret the results. As an example, which can be observed on Table 1, the sample of *St. Margarethen Limestone* treated with KSE 300 shows a porosity higher than the aged one, a treatment that is supposed to leave approximately 30% insoluble residue. Samples heterogeneity may be an explanation, but no definite elucidation has been found. As a consequence of this anomaly, all the values in Figure 4 result mostly uninformative and hard to interpret, especially for the range of pores larger than 10 µm.

Nevertheless, accepting the limitations of mercury intrusion porosimetry, we can still conclude that the major difference between the reference product KSE 300 and the NC products is the tendency of the NC consolidants to deposit in pores larger than 10 µm, which is especially well seen in *Schlaitdorf Sandstone*. Similar for all three consolidants is the deposition of silica gel in pores smaller than 10 µm. Even after the consolidation, pores in the range of 50–100 µm still represent the largest amount of pores present in the fabric. Treatment KSE 300 most likely indicates cracks in the silica gel, visible through an increase in pores approx. smaller than 0.01 µm, which were not there before. Moreover, treatments NC-25C and NC-27CP cause a disappearance of pores smaller than 0.05 µm, most likely due to the deposition of consolidants in these pores.

**Figure 5:**
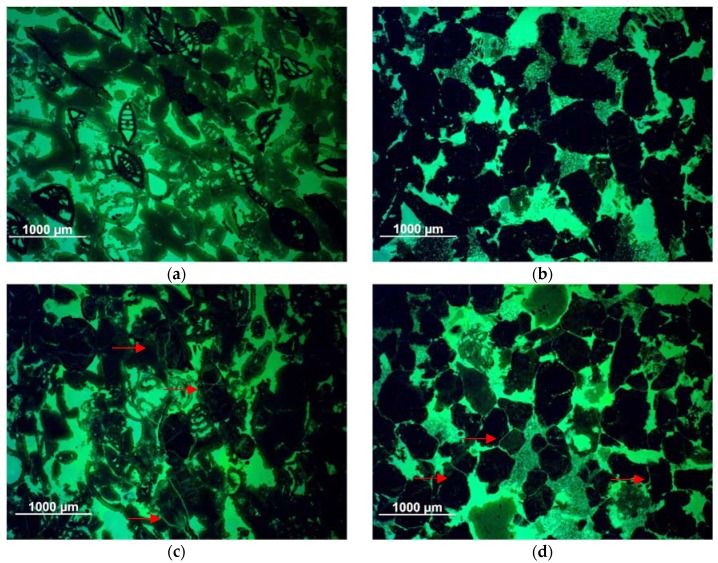
Microscopic views of thin sections made with a polarised-light microscope under UV-light using a fluorescent resin: (**a**) *St. Margarethen Limestone* in sound condition and (**c**) *St. Margarethen Limestone* in artificially aged condition displaying micro cracks mostly of an intra-granular nature passing through the microfossils; (**b**) *Schlaitdorf Sandstone* in sound condition and (**d**) *Schlaitdorf Sandstone* in artificially aged condition showing micro cracks mostly of an inter-granular nature affecting the grain boundaries.

*Schlaitdorf Sandstone* is relatively homogeneous, even in respect to the pore size distribution and geometry of the more or less equi-sized pores, and for this reason MIP values are more informative and easier to interpret. The artificial ageing created an evident shift in the pore radii distribution and an increase of the total open porosity. After ageing, and due to the pore shift, more pores larger than 10 µm and fewer pores smaller than 10 µm are present within the stone fabric when compared to the sound specimen. For both stones, PLM- and SEM studies are more appropriate to explain creations of micro cracks be it due to the artificial ageing or gel shrinkage (see Figure 5).

For *Schlaitdorf Sandstone*, the main modifications in Hg-porosimetry by treatments NC-25C and NC-27CP when compared to KSE 300 are striking (see the comparative graph in Figure 4). The key difference of NC-25C and NC-27CP products is their higher deposition in pores larger than 10 µm. For all three treatments, it can be concluded that the silica gel formed is deposited within a wide range of pores, only in different percentages. Furthermore, all treatments seem to produce a slightly higher amount of pores in the range of 5–10 nm, most probably due to the porosity of the gel shrinkage cracks.

In conclusion, a general tendency that can be extracted from the MIP measurements is that the NC products are able to fill the pores that govern and make up the largest percentage of porosity in the stone fabric and have thus the greatest impact on changes of the pore radii distribution. While all three consolidants are deposited in pores smaller than 10 µm, the capability of NC products to deposit also in larger pores seems to be the main cause for the increase in high mechanical strength, as explained below.

Comparing the values of mercury intrusion porosimetry for the studied stones, it can be concluded that each ageing protocol and the following treatment had their specific impact on the studied substrates. The treatments induce changes of the surface area, average pore size and geometry, pore radii distribution and total open porosity. All those alterations occur to different extents, pointing to the central role of the substrate in governing the effects. Not only are the consolidants different regarding their solid content after curing, but also the substrates capability to accommodate those products differs. However, even though the test results indicate measurable impacts on the physical stone parameters and hence the possible resistance towards weathering [61], potential consequences of those modifications are difficult to evaluate without predictive models and further studies in this area. In addition, the usefulness of this technique for certain types of stone needs to be evaluated with more precision and by statistical means. Until then, tests of mercury intrusion porosimetry will remain empirical and more of an indicative nature.

### 3.3. Evaluation of the Consolidation Efficiency (Mechanical Analysis)

#### 3.3.1. Effects of Thermal Treatment Prior to Consolidation

**Figure 6:**
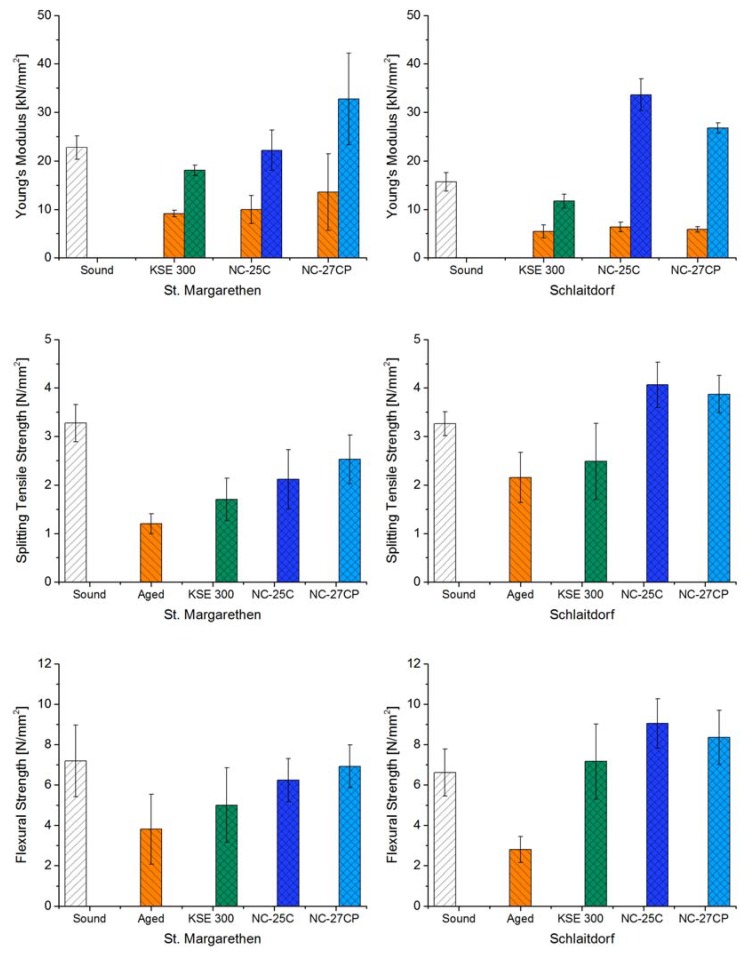
Illustration of Young’s modulus [kN/mm^2^], splitting tensile strength [N/mm^2^], and flexural strength under concentrated load [N/mm^2^] for *St. Margarethen Limestone* (**left side**) and for *Schlaitdorf Sandstone* (**right side**). All tests performed six months after consolidation. Please note that the dynamic modulus of elasticity was performed on the same specimens before and after treatment. Bars with orange filling represent aged samples before consolidation.

To assess the efficiency of stone consolidants, changes in strength and deformability (Young’s modulus) of sound, aged and consolidated specimens are the key parameters to be studied. The micro cracks induced by thermal ageing have caused a sufficient reduction of soundness in both lithotypes to study the capability of the consolidants to increase the mechanical strength of the samples. The decrease in soundness could be observed by all mechanical properties, namely by means of the dynamic Young’s modulus, splitting tensile- and flexural strength. However, the results differ in extent when comparing the lithotypes and test methods (Table 3 and Figure 6).

In the case of *St. Margarethen Limestone*, the thermal treatment reduced the soundness on average in terms of the dynamic Young’s modulus about 52% and 63% for *Schlaitdorf Sandstone.* Figure 5 displays fissures formed by thermal treatment. In *St. Margarethen Limestone*, the fissures are of a trans-granular type, i.e., crossing the biodetritic components, as opposed to *Schlaitdorf Sandstone*, where intergranular fissures affecting grain boundaries are present. Moreover, cracks presented in *Schlaitdorf Sandstone* seem to be more homogeneously distributed through the fabric when compared to *St. Margarethen*. The decrease in soundness determined through the Young’s modulus cannot provide direct information about the amount of cracks formed. The distribution of cracks and newly generated pores inside the fabric is also important since sound waves will propagate along the fastest traveling path. However, the Young’s modulus did reveal a greater deviation from the mean value in the case of *St. Margarethen Limestone*, which is in general an indication of a more inhomogeneous fabric in respect to the grainsize and pore radii distributions as comparison to *Schlaitdorf Sandstone*.

As for the splitting tensile test, strength decreased by ≈ 63% for *St. Margarethen Limestone* and by ≈ 34% for *Schlaitdorf Sandstone*. The flexural strength under concentrated load decreased by ≈ 47% for *St. Margarethen Limestone* and ≈ 58% for *Schlaitdorf Sandstone*. For both lithotypes, the absolute values determined by flexural strength are higher than corresponding properties measured by splitting tensile strength. For brittle materials, the main reason for such a behaviour is explained often with different volumes of material tested and the presence of material defects [62] (pp. 13–14). In other words, in larger samples the probability of defects density and distribution is higher, so the resultant values should be lower. However, Baumgartner [63] pointed out that more effort is necessary to distinguish which features may account for the differences in splitting tensile- and flexural strengths for natural stone. In general, the main factors influencing the indirect tensile strength values depend on the test method, the stone type with its scale and size effects, as well as the loading rate and testing procedure [64]. The actual tensile strength can be derived from indirect tests but only when the moduli ratio in tension and compression is known [65] or other measured properties like e.g., the crack initiation [66]. Moreover, to determine the influence of micro cracks also a closer look on the initial, elastic response of the stress-strain curves is necessary [67]. Such mechanical investigations, including simulations, would go beyond the scope of this study, but are desired in the field of stone conservation due to the microstructural heterogeneity of weathered and consolidated stone. However, even if at the moment closer interpretations are not part of this study, some conclusions can be drawn. Comparing both test methods, the anisotropy of the absolute average values for aged *Schlaitdorf Sandstone* is 0.76 and 0.31 for *St. Margarethen Limestone*. For *Schlaitdorf Sandstone*, this might be due to a more homogeneous distribution of cracks inside the fabric, while for *St. Margarethen Limestone*, the higher widespread of the absolute values indicate higher inhomogeneity and thus a strong influence of the sample geometry on the obtained values.

In any case, all test methods applied confirm the usefulness of the laboratory ageing prior to testing the efficiency of stone consolidants.

#### 3.3.2. Effects of Consolidation Treatment

All studied consolidation products induced a clear increase in the modulus of deformability (Young’s modulus) and strength. This phenomenon can be explained with the porous nature of both stones and the fissures induced through thermal aging, thus thanks to their ability to absorb and accommodate significant amounts of consolidants. Increases in Young’s modulus and strength have different impacts on the measured properties for the two lithotypes, with a tendency of *Schlaitdorf Sandstone* to show higher amount of increase (Table 3 and Figure 6). Increases followed a general trend for all three test methods employed with increments of KSE 300 < NC-25C < NC-27CP for *St. Margarethen Limestone* and KSE 300 < NC-27CP < NC-25C for *Schlaitdorf Sandstone*.

All three treatments had a great impact on the dynamic Young’s modulus. While KSE 300 exerts a similar effect for both lithotypes in respect to the relative increase of the modulus, for NC-25C and NC-27CP the modulus increased, respectively, two and three times more for *Schlaitdorf Sandstone* than for *St. Margarethen Limestone* (compare aged to consolidated condition in Table 3 and Figure 6). These latter treatments even raised the modulus to values above those recorded for the sound conditions of *Schlaitdorf Sandstone*–as a consequence of the better distribution of the silica gel in the newly formed fissures, clayey matrix and party also in the equi-sized voids, thus allowing a faster propagation of the elastic sound wave. The results cannot solely be explained through the solid content after curing since *Schlaitdorf Sandstone* accommodates less consolidant due to its less porous structure when compared to *St. Margarethen Limestone*. Here it becomes clear that the more intense crack network induced by ageing in *Schlaitdorf* and the given microstructure of the fabric have played a role in the treatment performance.

In general, there is no doubt that mineralogical, textural and structural features directly affect the strength of a stone. The same holds for the consolidated stone. Thus, the lithological and mineralogical composition, microstructural and microtextural features as well as petrophysical properties are the keys to understand deterioration processes and mechanical properties of lithotypes in macro scale. In view of this, the most likely reasons for why consolidation is more efficient for *Schlaitdorf Sandstone* than for *St. Margarethen Limestone*, as far as the strength increase is concerned, is the considerable amount of clay minerals and the equi-sized porosity. The non-swelling kaolinite is evenly distributed in the stone fabric, present as a filler component in the intergranular voids of this lithotype. After consolidation, clays seem to have been preferentially strengthened (Figure 2d), which probably helped to increase the mechanical strength above the sound condition. Clay minerals are known to be the governing factor influencing specific stone properties like e.g., sensitivity to weathering [68,69] or the hydro-mechanical behaviour with an extensive loss of strength [70]. In his book about alkoxysilanes and the consolidation of stone, Wheeler [24] (pp. 43–45) dedicated a section to the clay problem and consolidation. Herein he pointed out the contradictory results reported in the literature and the difficulty to draw definitive conclusions about consolidation efficiency of materials containing clays. However, numerous authors report successful treatment of clay bearing materials, for carbonate [24] (pp. 43–45, 54) and silicate varieties [71]. The issue of durability and potentially damaging consequences a consolidated clayey matrix may have is another question and, although not part of this study, it is worth further investigation.

Generally, the silicate substrate *Schlaitdorf* shows the highest consolidation effect and there are two possible reasons for that. The first reason is the often-mentioned concept of chemical affinity of alkoxysilane to silicate substrates. This means that the consolidation mechanisms in siliceous substrates are of a cohesive nature. It is explained by the formation of Si–O–Si bonds between consolidant and substrate [72]. In the case of the carbonate substrate, the consolidant is often referred to as a mere compacting agent, since it is deposited in the pore space of the fabric. There are two aspects that do not fit well in this concept: (i) in our study, the carbonate variety morphologically shows slightly better adhesion of the consolidant to the substrate, which was explained through the higher microrugosity of the grain interfaces. However, for both stones the adhesion was reported to be poor. In addition, (ii) since the beginning of its use and up to now, successful consolidation performances of alkoxysilanes have been reported for carbonate substrates in numerous studies [4,33,73,74,75,76,77,78,79]. Therefore, to explain better consolidation efficiency by solely referring to a chemical concept seems to be insufficient.

Consequently, the second possible reason for the higher effect of consolidation of the silicate substrate is the stone fabric itself. As stated above, the type of stone substrate plays the most significant role in both strength and deformability. Other authors have also pointed out that different fabrics and mineral compositions significantly influence the efficiency of stone consolidants [80,81]. Wheeler addressed the issue of structure by trying to consolidate larger quartz grains, which remained unconsolidated despite their chemical affinity [24] (pp. 46–48). It was pointed out that alkoxysilane gels tend to deposit in pores <50 µm and lesson their relative amount. The latter observation suggests that, depending on the deposition of the silica gel in certain pores representative for the studied fabrics, this will lead to efficiency in terms of mechanical strength. As can be extracted from the MIP data in the present study, the highest impact on pore radii can be observed for *Schlaitdorf Sandstone* treated with NC-25C and NC-27CP. Despite their influence on porosity due to the gel deposition rate, the unique property of the NC products is their capability to deposit also in larger pores, which make up the highest percentage in terms of open Hg-porosity (Figure 4). This phenomenon, together with the already described interpenetration of the clayey matrix located between the grains, form the main factors for the increase in mechanical strength.

Further studies are recommended to analyse the main factors (physico-mechanical features) influencing the efficiency of consolidation in terms of strength as well as the actual role of the chemical compatibility (chemi- and physisorption to the surface).

Regarding the practical conservation, the effect of *Schlaitdorfs* drastic increase in strength could result in the overconsolidation of surface layers known to cause harmful effects. In other words, to achieve a high mechanical strength through consolidation is not automatically linked to better performance of a treatment. Given the difficulty to produce a homogeneous in-depth distribution of any consolidant by surface treatment of an object, overconsolidation of certain layers can be hazardous. To avoid this risk, efforts are needed to optimize all controllable parameters related to a product and its mode of application for a lithotype in its given conditions. In stone consolidation, differences in strength and deformability are the most relevant parameters to assess. As thermal behaviour is critical in this respect, it should be included in further studies.

**Figure 7:**
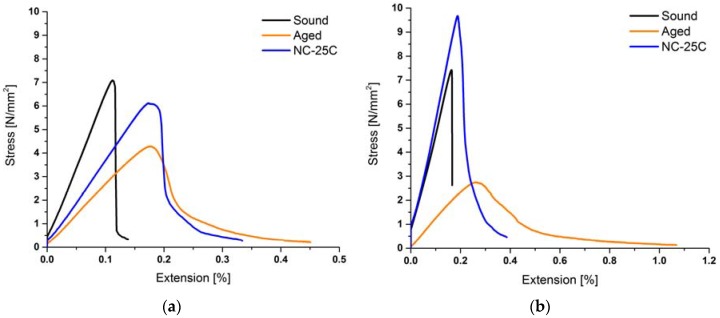
Examples of stress-strain curves exhibiting average values of the flexural strength under concentrated load. Sound, artificially aged, and subsequently consolidated conditions for (**a**) *St. Margarethen Limestone* specimens; and (**b**) *Schlaitdorf Sandstone* specimens, both tests performed six months after the consolidation treatment.

Regarding the mode of mechanical failure in the flexural strength test, it was observed that the sound stone can be categorized as brittle and the aged one rather as ductile (Figure 7). NC-25C has the capability to shift the failure mode from ductile back to quasi-brittle. Moreover, in the case of *Schlaitdorf Sandstone* this treatment restores the mechanical strength by exceeding the level of the sound stone condition. As the silica gel, formed after the curing of the consolidants, is a brittle material, there is no surprise that the stiffness is increased. On the other hand, the silica gel acts as a kind of filler, deposited inside the stone fabric, and reduces the absolute porosity hence the ductile deformability of the stone fabric is decreasing, which is why the failure mode is restored after the consolidation.

### 3.4. Evaluation of the Compatibility (Moisture Related Properties and Visual Impact)

In laboratory studies of compatibility, the main objective is to identify to what degree the stones’ physical properties change after treatment and to assess to which extent those changes may put the stone in jeopardy of an accelerated deterioration. While in the case of hydro-repellent treatments it is well known that some key physical parameters related to water transport get drastically changed. Within certain limits this is also observable for non-hydrophobic consolidation treatments. In this study, the two lithotypes were evaluated in respect to water absorption coefficient (WAC), water vapour permeability (WVP), and colour change. The respective measurements were performed for the lithotype samples in their sound, aged and consolidated conditions. Comparable to tests used to determine the efficiency, all results from the above-mentioned tests show changes of physical properties due to the treatments which, however, utterly differ between the studied lithotypes.

For both lithotypes, the micro cracks induced by ageing have increased the open porosity and correspondingly WAC (Table 4). After consolidation, the WAC is always reduced due to the presence of the consolidant residue in the stone fabric. However, consolidants such as TEOS, need an unknown period of time to fully react by hydrolysis and remain water repellent meanwhile, which results in an overestimation of their effect on WAC. Producers state that reaction times may last to approximately three to four weeks at 20 °C and 50% RH ambient conditions [82], but in laboratory conditions it appears that the system may stay water repellent for much longer time spans. In our study, the first set of tests were made after approximately 6 weeks, but it was considered necessary to repeat the WAC tests 6 months after the application of the consolidants. As evidenced by the results of Table 4 it appeared that for the products KSE 300 and NC-25C the rate of reaction accompanied by the loss of this temporary hydrophobicity not only differs between the products, but also between the substrates. Thus, the treatment KSE 300 does not affect the WAC in the case of *St. Margarethen Limestone* but it does affect *Schlaitdorf Sandstone* resulting in a water repellent surface even after 6 weeks of curing. On contrary, NC-25C does affect the WAC in the same manner within *St. Margarethen Limestone* but does not affect *Schlaitdorf Sandstone*. At the present stage, it is not clear which of the stone parameters influences the reaction rate of TEOS. Measurements taken six months after the application are stabilized and reveal a probable end state of the curing process. This indicates the importance of the timing of the test protocol and points out that even mechanical parameters would evolve within the prolonged periods of curing. These observations are particularly relevant to on-site follow up of restoration and conservation activities.

As for the treatment NC-27CP, a consolidant with explicitly hydrophobic components, its lasting water repellence could be proved by the low WAC values even after 6 months of curing. The WAC values can be classified as water repellent and water hindering [83] (p. 215). The slight differences in the 6 months WAC of this product between both lithotypes is also confirmed by the contact angles of water—96.78° for *Schlaitdorf Sandstone* and 87.26° for *St. Margarethen Limestone*. In the case of *St. Margarethen Limestone* the WAC can be classified as water hindering as it is higher than the referenced threshold (<0.5 kg·m^−2^·h^−0.5^). Moreover, it is also slightly below the threshold for the contact angle of water of 90° as reported in some studies [84,85].

The drying properties of consolidated materials are important and are recommended to be analysed further as they can give more insights into potentially damaging consequences.

As regards water vapour permeability (WVP), its drastic reduction is not advantageous for several reasons like, e.g., water trapping and subsequent contour scaling, decreased drying rate and thus mobilization of salts or advancement of bio-growth, etc. Different thresholds (between 5 and 20%) are reported in literature related to an acceptable percentage of decrease in WVP [86,87]. Since in the present study this test was performed only 6 weeks after the treatment of the specimens, the state of progress of the gel forming reaction and hence the temporary water repellency were undefined, possibly under- or overestimating the WVP after consolidation (Table 4). The reason for the behaviour of KSE 300 on *Schlaitdorf Sandstone* is unknown and the increase in WVP after the treatment needs further clarification. On the other hand, increased WVP values following a water repellent treatment have been reported for some cases [88,89]. Generally, it must be assumed that any treatment would have an impact on the polar properties of the mineral surfaces of a porous body and hence influence the wettability in one or the other way. WVP values of KSE 300 in the case of *St. Margarethen Limestone* are reasonably close to the untreated stone and thus a low impact in the overall incompatibility may be anticipated. Changes reported for treatment NC-25C are <20% and display thus the most promising results for both lithotypes. An interesting phenomenon that can be observed within *St. Margarethen Limestone* is that WAC is drastically affected by the rate of the solid content after curing (differences between 30% and 50%) while the corresponding WVP values remain almost identical.

Nano titania exhibits super hydrophilicity when it absorbs a photon with energy equal or greater than its band-gap (3.0–3.2 eV) [90]. At the moment, this property cannot be distinguished from the hydrophilicity of the silica gel. For such purposes, the same consolidant should have been tested with and without nano titania. The eventual hydrophilic properties of nano titania induced by exposure to UV light will be investigated in subsequent research. The differences in capillary water absorption and water vapour permeability of consolidated stone can at the present stage be attributed to the different amount of solid content after curing, rather than to a hydrophilic behaviour induced by nano TiO_2_.

The hydrophobic treatment NC-27CP shows reductions of WVP ranging from 45% for *St. Margarethen Limestone* to 65% for *Schlaitdorf Sandstone*. This eventually corresponds to a higher impact in respect to incompatibility and related risks, even though no distinct threshold can be stated for all conditions. In addition, numerous authors have reported that the effectiveness and compatibility of a water repellent treatment depends on the substrate under study [91,92,93]. What is usually studied in regards to hydrophobic treatments is their durability and subsequent loss of water repellency [94,95] but not the potentially damaging consequences of the treatment. Potential damaging consequences would include studies related to water transport and undesired condensation phenomena, vapour pressure and corresponding temperature differences, comparable to reports obtained elsewhere [96].

For the study of alkoxysilanes, further determination of WVP measurements at different time intervals is desired to better understand the delay due to the chemical reaction. This might be of great importance for on-site conditions where larger penetration depths play a major role and the kinetics of the reaction are often complicated by different contaminants. Moreover, a study of different decay patterns (e.g., biological growth, black crusts, profile of degradation, etc.) in regards to their WVP is also recommended to further analyse the effect of consolidants on-site.

The values regarding colour measurements taken six weeks and one year after consolidation are summarized in Table 5. It was observed that even under laboratory conditions, the colour changes over time due to the chemical reaction and not following the same trends as found in the field. In fact, some values increase while others decrease from 6 weeks to 12 months’ time interval. By now, it is clear that the studied substrates affect differently the treatments performance. As a general overview, colours tend to evanesce with time, more systematically in the silicate substrate, coming close to a value of ΔE* < 5, normally accepted as a threshold of perceptible colour impacts [11,97]. The impact was higher in the carbonate substrate and its evolution in time was less favourable.

As usually happens in stone consolidation, the studied products have modified the stone properties, and some modifications signify that they may be responsible for a certain degree of incompatibility, in terms of the methodology proposed by Delgado Rodrigues and Grossi [11]. Given the specific character of this research, a more thoughtful application of that methodology was not considered. However, it needs to be emphasized that the values reported for the compatibility assessment should not be considered as solely entities that must satisfy a rigid requirement. A compatibility assessment, that would include also tests of durability, is a result of multivariate processes that requires the treatment of several parameters simultaneously to end up with an overall risk evaluation. That is to say, not meeting a threshold of one of the above studied parameters cannot result in excluding a consolidation treatment especially after the gained results in this study clearly indicate a wide-ranging evolution of values in time and magnitude.

## 4. Conclusions

This study addressed the potential efficiency and compatibility of three alkoxysilanes on two substrates of different chemo-mineralogical and petrophysical properties, namely a carbonate and a silicate stone. Results clearly show that treatment performance depends primarily on the textural and microstructural parameters of the stone fabric. Under the same conditions, the same consolidant applied on different substrates yielded differences in mechanical and physical properties.

For the studied lithotypes, there is a clear trend observed within all three test methods to evaluate the efficiency. The relative percentage of deformability (Young’s modulus) and strength increase, and the absolute values achieved, differ between the products applied and the lithotypes studied. The tendencies of *Schlaitdorf Sandstone* to show higher increases in deformability modulus and strength are related to its fabric. SEM studies provided insights into the preferential consolidation and interpenetration of kaolinite that is homogeneously located in intergranular pores inside the fabric. MIP analyses showed that the NC products not only deposit in smaller pores were the clay packs play an important role but also in large pores that actually make up the leading pore ranges in terms of open Hg-porosity. Therefore, it seems likely that the interpenetration of the clayey matrix combined with the deposition of the consolidants in all pore ranges, causing the most pronounced shift in the pore radii distribution, resulted in a mechanical strength above the values of the original stone material.

In what concerns the mechanical strength of the carbonatic lithotype *St. Margarethen Limestone*, the treatments show a sufficient increase in strength and deformability but the fabrics inhomogeneity with large voids and intragranular cracks passing though the microfossils did not allow the increase to be as high as the one described for *Schlaitdorf Sandstone*. Moreover, the main pore radii, which makes up the largest percentage of the open Hg-porosity in *St. Margarethen Limestone* seemed not to be filled to the same degree with the silica gels as observed within *Schlaitdorf Sandstone*.

In regards to the strength parameters, the experimental data also show that the amount of a consolidant deposited after curing has a relevant contribution for the overall consolidation action, even if its incidence is not fully proportional, as seen by the lower increments in deformability and strength caused by NC-27CP in *Schlaitdorf Sandstone* when compared to NC-25C. The reason for such behaviour needs to be further studied.

Despite the differences in strength increase, all consolidants show a strengthening action, therefore fulfilling their intended purpose.

The physical changes induced by treatments showed to be dependent on the time span after application. The necessary duration needed to obtain stable physical properties is difficult to predict and may primarily depend on the amount of consolidant deposited inside the stone’s fabric and are therefore substrate dependent. Relating to water absorption by capillarity recorded six weeks after application, the temporary hydrophobicity of KSE 300 lasts for longer time in the silicate substrate but it does not affect the carbonate substrate. On the contrary, treatment NC-25C affects the carbonate substrate in terms of six weeks hydrophobicity but not the silicate substrate. More research is needed to analyse which stone parameters influence the reaction rate of TEOS. In respect to the substrate, the evolution values in time, for WVP and colour, follow no general trend and display opposing directions varying in absolute and relative magnitude.

It can be concluded that even with a system like alkoxysilanes, which have been in the focus of scientific interest for decades, major differences in compatibility are evident. In this study, it was demonstrated that for such reactive systems, an evaluation within the first months is not representative and yields an over- or underestimation of parameters. This study clearly demonstrated how widespread the gained results are when one system is applied on different substrates, both in time and relative and absolute magnitude.

To tailor the performance of a treatment, an experimental study prior to field application seems unavoidable. This is in order to understand the relationship between substrate and treatment, since all evaluated products, the newly engineered and the reference material, react unpredictably and depend primarily on the substrate. Only with the analysis of several parameters simultaneously, is an overall risk evaluation possible.

## Figures and Tables

**Table 1:** Results from mercury intrusion porosimetry for specimens in sound, aged and consolidated conditions for *St. Margarethen Limestone* and *Schlaitdorf Sandstone*.

Porometric Characteristics of Materials		Conditions				
		Sound	Aged	KSE 300	NC-25C	NC-27CP
***St. Margarethen Limestone***	Total pore surface [m^2^/g]	0.824	1.326	2.168	0.523	0.569
	Average pore diameter [µm]	0.483	0.253	0.183	0.526	0.568
	Total porosity [%]	20.65	17.53	20.63	14.76	16.88
***Schlaitdorf Sandstone***	Total pore surface [m^2^/g]	0.800	0.518	0.477	0.864	0.242
	Average pore diameter [µm]	0.349	0.643	0.533	0.223	0.463
	Total porosity [%]	15.42	18.02	14.20	11.15	7.10

**Table 2:** Gel deposition rate or solid content of cured consolidants calculated gravimetrically on three specimens per stone and treatment after 4 weeks of curing. As already mentioned in the text, the values reported may not be definite because of the incomplete reaction at this age.

Product Specification	*St. Margarethen Limestone*			*Schlaitdorf Sandstone*		
Consolidant	KSE 300	NC-25C	NC-27CP	KSE 300	NC-25C	NC-27CP
Solid Content [%]	31.62	47.82	59.73	32.12	46.59	59.98
Standard Deviation	±1.46	±0.57	±0.54	±1.04	±0.67	±0.50

**Table 3:** Rounded values for relative decrease/increase [%] for sound (S), aged (A) and consolidated (C) samples and absolute average values of Young’s modulus [kN/mm^2^], splitting tensile strength [N/mm^2^], and flexural strength under concentrated load [N/mm^2^] for *St. Margarethen Limestone* and *Schlaitdorf Sandstone*. The graphical visualisation of the same values can be found in Figure 6. All tests were performed six months after consolidation.

Consolidant			(S) Sound ± Std.N	(A) Aged ± Std.N	(C) Consolidated ± Std.N	Decrease (S-A, %)	Increase (A-C, %)	Magnitude (S-C, %)
***St. Margarethen Limestone***	Young’s Modulus (kN/mm^2^)	KSE 300	22.7 ± 2.4	9.2 ± 0.7	18.1 ± 1.1	−60	+97	−21
		NC-25C		10.0 ± 2.9	22.2 ± 4.1	−56	+123	−2
		NC-27CP		13.6 ± 7.9	32.7 ± 9.5	−40	+141	+44
	Splitting Tensile Strength (N/mm^2^)	KSE 300	3.3 ± 0.4	1.2 ± 0.2	1.7 ± 0.4	−63	+42	−48
		NC-25C			2.1 ± 0.6		+76	−35
		NC-27CP			2.5 ± 0.5		+111	−23
	Flexural Strength (N/mm^2^)	KSE 300	7.2 ± 1.8	3.8 ± 1.7	5.0 ± 1.8	−47	+31	−31
		NC-25C			6.2 ± 1.1		+64	−13
		NC-27CP			6.9 ± 1.1		+82	−4
***Schlaitdorf Sandstone***	Young’s Modulus (kN/mm^2^)	KSE 300	15.7 ± 1.9	5.5 ± 1.3	11.7 ± 1.4	−65	+114	−26
		NC-25C		6.4 ± 1.0	33.6 ± 3.3	−60	+429	+114
		NC-27CP		5.9 ± 0.6	26.8 ± 1.1	−63	+356	+70
	Splitting Tensile Strength (N/mm^2^)	KSE 300	3.2 ± 0.2	2.2 ± 0.5	2.5 ± 0.8	−34	+16	−24
		NC-25C			4.1 ± 0.5		+89	+25
		NC-27CP			3.9 ± 0.4		+80	+19
	Flexural Strength (N/mm^2^)	KSE 300	6.6 ± 1.2	2.8 ± 0.6	7.2 ± 1.9	−58	+156	+8
		NC-25C			9.0 ± 1.2		+223	+37
		NC-27CP			8.4 ± 1.3		+198	+26

**Table 4:** Values of water absorption coefficient after one-hour (WAC) [kg·m^−2^·h^−0.5^] and ratio of water vapour permeability values (WVP c/a) [kg·m^−1^·s^−1^·Pa^−1^], for sound (s), aged (a) and consolidated (c) specimens of *St. Margarethen Limestone* (SM) and *Schlaitdorf Sandstone* (S) evaluated six weeks (6w) and six months (6 m) after treatment.

Stone	Treatment	WAC(s)	WAC(a)	WAC(c) (6 w)	WAC(c) (6 m)	WVPc/a (6 w)
SM	KSE 300	4.49 ± 0.05	5.23 ± 0.02	4.52 ± 0.06	4.56 ± 0.03	0.89 ± 0.06
	NC-25C	4.50 ± 0.18	5.05 ± 0.11	0.76 ± 0.27	2.99 ± 0.28	0.84 ± 0.03
	NC-27CP	4.51 ± 0.15	5.17 ± 0.18	0.47 ± 0.51	0.54 ± 0.26	0.55 ± 0.02
S	KSE 300	3.29 ± 0.30	3.66 ± 0.28	0.18 ± 0.15	2.14 ± 0.50	1.75 ± 0.45
	NC-25C	2.74 ± 0.72	3.57 ± 0.25	1.65 ± 0.15	1.21 ± 0.28	0.81 ± 0.03
	NC-27CP	2.74 ± 0.59	3.53 ± 0.16	0.08 ± 0.04	0.20 ± 0.04	0.35 ± 0.00

**Table 5:** Colour values measured on sound *St. Margarethen Limestone* (SM) and *Schlaitdorf Sandstone* (S) before and after treatment at different intervals: six weeks (6 w) and twelve months (12 m).

Stone	Treatments	ΔL* (6 w)	ΔL* (12 m)	Δa* (6 w)	Δa* (12 m)	Δb* (6 w)	Δb* (12 m)	ΔE* (6 w)	ΔE* (12 m)
SM	KSE 300	4.51	6.62	0.87	0.45	5.30	4.53	7.01	8.03
	NC-25C	0.87	2.71	1.29	0.92	5.15	3.98	5.38	4.90
	NC-27CP	−1.41	0.44	2.10	1.51	7.31	6.15	7.74	6.35
S	KSE 300	−5.49	2.46	1.10	0.81	5.80	3.84	8.06	4.63
	NC-25C	0.30	2.77	0.76	1.39	2.86	5.04	2.97	5.92
	NC-27CP	−2.57	1.63	0.61	0.75	0.76	0.66	2.75	1.91

L* the lightness coordinate ranging from 0 (black) to 100 (white), with positive values meaning darkening; a* the red/green coordinate, with +a* indicating redness and –a* indicating greenness; and b* the yellow/blue coordinate, with +b* indicating yellowness and –b* indicating blueness.
